# Mutant hFGF23(A12D) stimulates osteoblast differentiation through FGFR3

**DOI:** 10.1111/jcmm.14201

**Published:** 2019-02-13

**Authors:** Yilin Tu, Taoran Qu, Fengshan Chen

**Affiliations:** ^1^ Laboratory of Oral Biomedical Science and Translational Medicine, School and Hospital of Stomatology Tongji University Shanghai China

**Keywords:** bone biology, bone remodelling/regeneration, cell proliferation, cell signalling, fibroblast growth factor 23, mandibular prognathism

## Abstract

Fibroblast growth factor (FGF) 23 is a member of the FGF family involved in bone development by interacting with FGFRs. In a previous study, we discovered a mutant human FGF (hFGF) 23 (A12D) in the mandibular prognathism (MP) pedigree. However, the exact role of hFGF23(A12D) during bone formation remains unclear. The aim of this study was to identify the function of hFGF23(A12D) in bone formation. We infected isolated rat calvaria (RC) cells with the recombinant lentivirus containing mutant hFGF23(A12D) and WT hFGF23 respectively. Real‐time PCR, western blot and enzyme‐linked immunosorbent assay confirmed that hFGF23(A12D) failed to be secreted. We measured cell growth via the CCK‐8 assay based on Zsgreen expression, detected cell differentiation ability via alkaline phosphatase staining, performed RT‐PCR and found that hFGF23(A12D) inhibited proliferation of RC cells and stimulated the differentiation of RC cells to osteoblasts. Through RNA sequencing, RT‐PCR and western blot, we found increased expression of FGFR3. Through co‐immunoprecipitation assays and immunofluorescence staining, we revealed that hFGF23(A12D) activated the mitogen‐activated protein kinase signalling pathway through interactions with the intracellular domain of FGFR3. In summary, we determined the mechanisms of hFGF23(A12D) involved in osteoblast generation and formation which is specifically due to its interaction with FGFR3.

## 1 INTRODUCTION

Fibroblast growth factor (FGF) 23 is a member of the FGF19 subfamily of FGFs, which is mainly secreted by osteocytes and osteoblasts, and is part of the systemic circulation.^1^, ^2^ Human FGF23 is a 32 kD protein, which consists of 251 amino acids and includes a signal peptide composed of 24 amino acids in the N‐terminal portion of the protein^3^ that works as an inhibitor of bone mineralization.^4^, ^5^ FGF23 can suppress phosphate reabsorption and 1,25(OH)2D in the kidney,^6^, ^7^ and FGF23 null mice show high serum phosphate levels with increased renal phosphate reabsorption and growth retardation with an abnormal bone phenotype.^8^ Moreover, FGF23 treatment in rat calvaria (RC) cells and MC3T3.E1 can negatively regulate both differentiation and matrix mineralization through the interaction of FGF23 with FGFR1c.^9^, ^10^ The data suggest that FGF23 functions as an endocrine factor and has direct effect on bone.

Mandibular prognathism is a maxillofacial disorder characterized by overgrowth of the mandibular bone.^11^ Our previous study reported genome‐wide linkage and whole‐exome sequencing analyses on an MP pedigree, and in three unrelated MP patients, we discovered a mutation of FGF23 c.35C>A.^12^ The treatment of mandibular prognathism in clinical includes orthodontic therapy and orthognathic surgery. Both of them involve bone remodelling which lead us to consider how mutant hFGF23 would affect bone remodelling during treatment.

In this study, we used osteoblasts to evaluate the direct function of FGF23 (A12D) in bone development. We determined that hFGF23(A12D) inhibited proliferation of RC cells and stimulated the differentiation of RC cells to osteoblasts through activating the mitogen‐activated protein kinase (MAPK) signalling pathway via interactions with the intracellular domain of FGFR3.

## 2 MATERIALS AND METHODS

### 2.1 Lentiviral construction and preparation

The open reading frame (ORF) of wild or mutant human FGF23 was cloned into the pLVX‐mCMV‐ZsGreen plasmid (Viraltherapy Technologies, Wuhan, China). Subsequently, lentiviruses (rLV‐hFGF23(A12D)‐mCMV‐ZsGreen and rLV‐hFGF23‐WT‐mCMV‐ZsGreen) were prepared by transfection of plasmids (pLVX‐hFGF23‐mCMV‐ZsGreen and pLVX‐hFGF23(A12D)‐mCMV‐ZsGreen) into 293 T cells. Lentivirus (rLV‐mCMV‐ZsGreen) was used as a control.

### 2.2 Isolation, infection and induction of RC Cells

Male Sprague Dawley (SD) rats were purchased from the Shanghai SLAC Laboratory Animal Co. Ltd. Primary RC cells were isolated from 24‐hour‐old SD rats. Briefly, calvariae were dissected and sectioned into fractions. After digestion with type II collagenase (Sigma‐Aldrich, St. Louis, MO, USA) for 1 hour, calvariae fractions were placed into 6 cm cell culture dishes containing alpha‐modified Eagle's medium (α‐MEM; Hyclone, Logan, UT, USA) supplemented with 12% foetal bovine serum (Gibco, Grand Island, NY, USA) and 1% penicillin/streptomycin. The third passages of RC cells were trypsinized and cultured at 0.5 × 10^4^/cm^2^ in the same medium. When cells reached 70% confluency, they were infected with three lentiviruses (rLV‐hFGF23(A12D)‐mCMV‐Zsgreen, rLV‐hFGF23‐WT‐mCMV‐Zsgreen and rLV‐mCMV‐ZsGreen) at a multiplicity of infection of 60 pfu/cell in the same medium containing 5 μg/mL polybrene (Sigma‐Aldrich). Some cells were infected on day 15.

Rat calvaria cells infected with lentivirus were cultured in α‐MEM supplemented with 10% FBS and 1% P/S additionally with 50 μmol/L ascorbic acid (Sigma‐Aldrich), 10 mmol/L β‐glycerophosphate (Sigma‐Aldrich) and 100 nmol/L Dexamethasone (Sigma‐Aldrich). In some cultures, SCH772984, SB203580 and AZD4547 (Selleck Chemicals, Houston, TX, USA) or vehicle (DMSO) were added from the fifth day.

### 2.3 Enzyme‐linked immunosorbent assay

After the cells were infected and osteogenically induced, conditioned media were collected and stored at −80°C until use. FGF23 was determined using the human FGF23 enzyme‐linked immunosorbent assay (ELISA) kit (BiotechWell, Shanghai, China), according to the manufacturer's instructions.

### 2.4 Cell proliferation assay

Cell proliferation was measured using the Cell Counting Kit‐8 (Beyotime, Shanghai, China). Briefly, cells were trypsinized and cultured in a 96‐well plate (0.3 × 10^4^/cm^2^) and then infected with rLV‐mCMV‐ZsGreen, rLV‐hFGF23(A12D)‐mCMV‐ZsGreen and rLV‐hFGF23‐WT‐mCMV‐ZsGreen. Cells were incubated with 10 µL CCK‐8 per well for 1 hour and then measured at a 450 nm wavelength using an absorbance reader (ELx800; Bio‐tek Inc, Winooski, VT, USA).

### 2.5 Alkaline phosphatase staining and quantification

Alkaline phosphatase staining was performed using the Alkaline Phosphatase Staining Kit (Beijing Leagene Biotechnology, Beijing, China), according to the manufacturer's instructions. Images were captured, and the number of ALP+ cells in each well was counted using a stereomicroscope (EVOS; Thermo Fisher Scientific, Inc, Beijing, China).

The quantity of alkaline phosphatase was evaluated by the Alkaline Phosphatase Assay Kit (Nanjing Jiancheng, Nanjing, China), and the total protein concentration was determined using a BCA protein assay kit (Beyotime), according to the manufacturer's instructions.

### 2.6 Alizarin red staining

Cells were stained in Alizarin red staining solution (Cyagen Biosciences, Suzhou, China), according to the manufacturer's instructions, and images were taken with a stereomicroscope. Subsequently, cells were submerged in 1 mL 10% hexadecylpyridinium chloride for 30 minutes at 37°C. Finally, a microplate reader was used to measure the absorbance at 562 nm.

### 2.7 RNA extraction and real‐time PCR analysis

Total RNA was extracted using the TRIzol reagent (Invitrogen, Carlsbad, CA, USA), according to manufacturer's instructions. After detection of the concentration and purity of total RNA with the spectrophotometer (Nanodrop, Thermo Fisher Scientific), the RNA was reverse‐transcribed to cDNA using a Prime‐Script RT reagent kit (Takara Bio, Dalian, Japan) at 37°C for 15 minutes, then 85°C for 5 seconds. Real‐time PCR was performed with the Applied Biosystems 7300 Real‐Time PCR Systems (Thermo Fisher Scientific) using SYBR green (SuperReal PreMix Plus, SYBR Green; TIANGEN, Beijing, China), according to the manufacturer's directions. Primers (Table 1) were synthesized commercially (Sangon, Shanghai, China). The products were verified by melting curve analysis, and the relative gene expression levels were calculated by the 2^−ΔΔCT^ method.

**Table 1:** Primer sequence for real‐time PCR

Gene	Primers
ALP‐F	TCCCAAAGGCTTCTTCTTGC
ALP‐R	ATGGCCTCATCCATCTCCAC
BMP‐2‐F	GAAGCCAGGTGTCTCCAAGAG
BMP‐2‐R	GTGGATGTCCTTTACCGTCGT
COLI(α)‐F	CTGCCCAGAAGAATATGTATCACC
COLI(α)‐R	GAAGCAAAGTTTCCTCCAAGACC
FGF23‐F	TTGGATCGTATCACTTCAGC
FGF23‐R	TGCTTCGGTGACAGGTAG
FGFR3‐F	AGTGTGCGTGTAACAGATG
FGFR3‐R	AGCCAGCAGTTTCTTATCC
GAPDH‐F	GACCCCTTCATTGACCTCAACTA
GAPDH‐R	AAGTTGTCATGGATGACCTTGGC
OCN‐F	GCCCTGACTGCATTCTGCCTCT
OCN‐R	TCACCACCTTACTGCCCTCCTG
OPN‐F	CTGCCAGCACACAAGCAGAC
OPN‐R	TCTGTGGCATCGGGATACTG

### 2.8 RNA sequencing

Total RNA was extracted with the TRIzol reagent on the ninth day of osteogenic induction. First, we checked total RNA quality. Subsequently, mRNA was purified with oligo‐dT, and the retrieved RNA was fragmented to obtain 100‐300 bp fragments. The first and second cDNA strands were synthesized. Double‐strand cDNA was purified with magnetic beads and resolved in elution buffer for end repair and adenylated 3′ ends. After ligating adapters and performing size selection, the products were amplified to build the cDNA library. Sequencing was performed by the Illumina Hiseq 2500 (Illumina Inc, CA, USA) after a library quality check. Cuffdiff software was used to analyse the differential expression, and *P < *0.05, |log2 (fold change)|>1 and *q* value <0.05 were considered significant.

### 2.9 Bioinformatics prediction

The secondary structures of hFGF23(A12D), hFGF23‐WT and FGFR3 were predicted with the website Bloomsbury Centre for Bioinformatics group (http://bioinf.cs.ucl.ac.uk/introduction/). The tertiary structures of hFGF23(A12D) and FGFR3 protein were predicted with the website Swiss‐model (https://www.swissmodel.expasy.org/). The binding affinities of hFGF23(A12D) and the intracellular domain of FGFR3 were predicted with the website PPA‐Pred2 (http://www.iitm.ac.in/bioinfo/PPA_Pred/prediction.html).

### 2.10 Transient transfection of 293 T cells

The ORFs of mutant hFGF23(A12D) with the HA‐tag and FGFR3 (Protein Kinases; catalytic domain, PKCD) with the MYC‐tag were cloned into a pcDNA3.1 plasmid (Viraltherapy Technologies).

Briefly, 293 T/RC cells were grown in a 10 cm cell culture dish, and transient transfection was performed using Lipofectamine™ 2000 Transfection Reagent (Thermo Fisher Scientific), according to the manufacturer's instructions. Each dish was transfected with 12 µg of HA‐FGF23(A12D)/pcDNA3.1‐HA and MYC‐FGFR3(PKCD)/pcDNA3.1‐MYC. Cells were collected after 48 hours.

### 2.11 Immunofluorescence staining

Cells were washed three times with cold PBS and fixed in 4% paraformaldehyde for 15 minutes. Subsequently, cells were washed three times with PBS and incubated with 0.2% Triton X‐100 (Beyotime) for 20 minutes. After washing three times in PBS, cells were incubated with 1% BSA for 30 minutes at room temperature. The cells were then incubated overnight at 4ºC with the following antibodies: rabbit anti‐FGFR3 (1:50; Abcam Inc, Cambridge, MA, USA), rabbit anti‐HA tag (1:25; Cell Signaling, Beverly, MA, USA) and mouse anti‐MYC tag (1:25; Cell Signaling). Following this, cells were washed with PBST three times and were incubated with secondary antibodies (Donkey‐anti‐rabbit, Abcam Inc; Donkey‐anti‐mouse, Abcam Inc) for 1 hour at 37ºC. Finally, the cells were washed with PBST and observed under a Laser scanning confocal microscopy (Nikon A1R, Tokyo, Japan).

### 2.12 Co‐immunoprecipitation

Cells were lysed in cold immunoprecipitation (IP) buffer containing a protease inhibitor (BiotechWell). After centrifugation, supernatants were collected, and 20 µL of Protein A/G was added (Santa Cruz Biotech, Dallas, TX, USA). After incubation at 4ºC for 30 minutes, supernatants were collected and incubated with HA‐tag/MYC‐tag antibodies (Cell Signaling) at 4ºC overnight. Subsequently, 50 µL of Protein A/G was added and samples were incubated at 4ºC for 6 hours. Samples were washed five times with IP buffer and resuspended in 60 µL of 2× electrophoresis sample buffer.

### 2.13 Western blotting

Cells were lysed in cold RIPA buffer with protease inhibitors (BiotechWell). Proteins were separated by SDS‐PAGE and transferred onto PVDF membranes. Membranes were blocked using 5% BSA in TBST and incubated overnight at 4ºC with the following antibodies: rabbit anti‐HA tag (1:2000), mouse anti‐MYC tag (1:2000), goat anti‐FGF23 (1:1000) and rabbit anti‐FGFR3 (1:1000). After washing three times with TBST, membranes were incubated for 1 hour at room temperature with secondary antibodies. Finally, membranes were washed three times with TBST, and data were captured with a chemiluminescence detection system (GE AI600, Boston, MA, USA).

### 2.14 Statistics analysis

All data were analysed by one‐way ANOVA and independent *t* tests. *P < *0.05 was considered significant.

## 3 RESULTS

### 3.1 Exogenous mutant hFGF23(A12D) was overexpressed in RC cells and failed to be secreted

**Figure 1:**
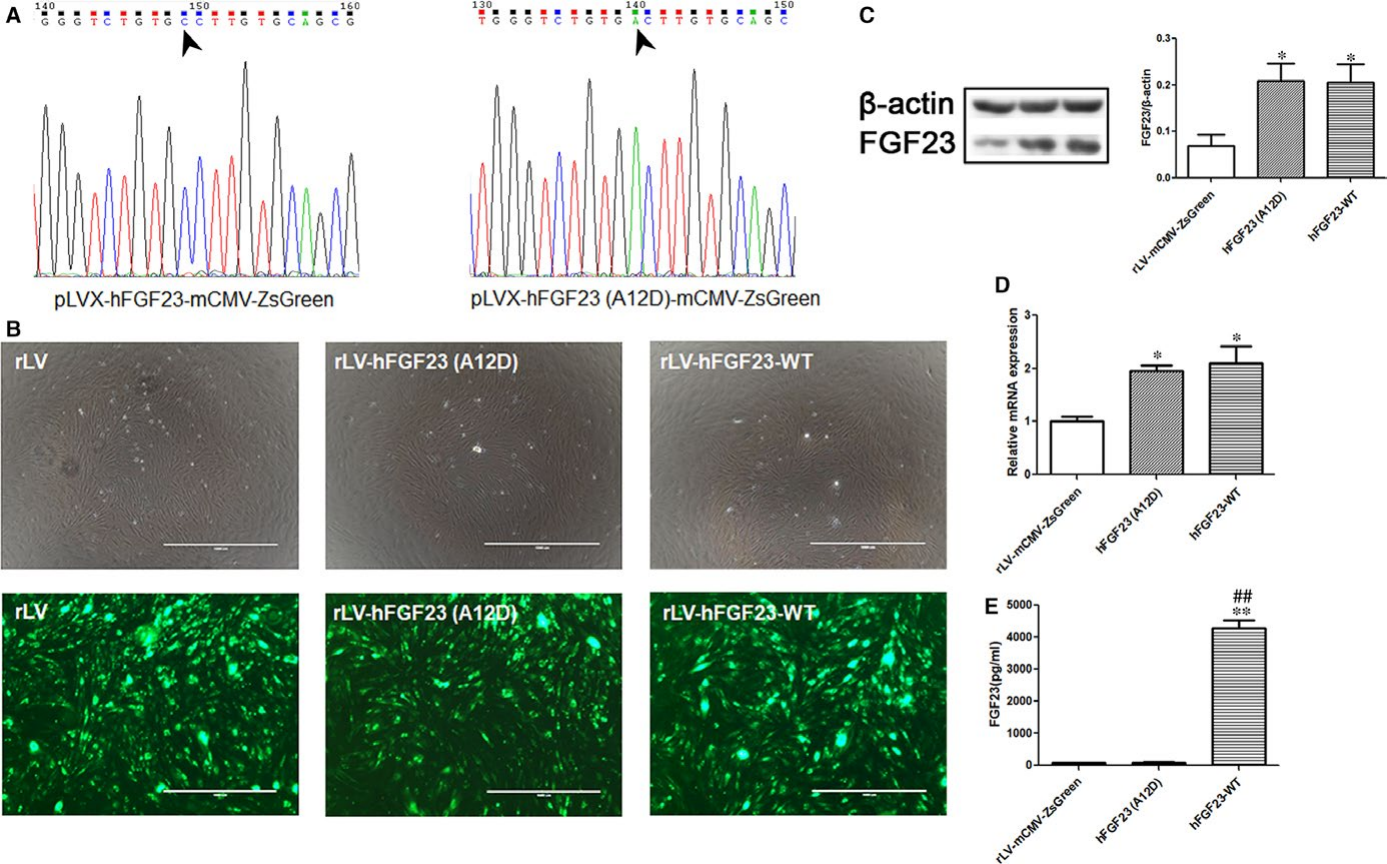
Exogenous mutant hFGF23(A12D) is overexpressed in RC cells and fails to be secreted. A, Sequencing result confirmed the correction of pLVX‐hFGF23‐mCMV‐ZsGreen and pLVX‐hFGF23‐A12D‐mCMV‐ZsGreen. B, The fluorescence image shows the infection efficiency of the three lentiviruses. C, Western blot and (D) RT‐PCR results show that hFGF23 is overexpressed both at protein level and mRNA level respectively. E, ELISA result shows the expression of secreted hFGF23 in supernatants. *P < 0.05 and **P < 0.01 vs control group. ^##^ *P < *0.01 vs hFGF23(A12D) group. Values are presented as mean ± SD fold change

To validate whether mutant hFGF23(A12D) affects osteoblast genesis, we isolated RC cells, osteogenic progenitor cells, from SD rats. First, we constructed pLVX‐hFGF23‐mCMV‐ZsGreen and pLVX‐hFGF23(A12D)‐mCMV‐ZsGreen and obtained the correct insert sequences (Figure 1A). We also prepared recombinant lentiviruses rLV‐hFGF23(A12D)‐mCMV‐ZsGreen and rLV‐hFGF23‐WT‐mCMV‐ZsGreen. RC cells were infected by rLV‐mCMV‐ZsGreen, rLV‐hFGF23(A12D)‐mCMV‐ZsGreen and rLV‐hFGF23‐WT‐mCMV‐ZsGreen. We calculated the ZsGreen fluorescence ratio of rLV‐mCMV‐ZsGreen, rLV‐hFGF23(A12D)‐mCMV‐ZsGreen and rLV‐hFGF23‐WT‐mCMV‐ZsGreen via microscope in order to evaluate the infection efficiency of the three lentiviruses (Figure 1B). As shown in Figure 1C and D, overexpression of hFGF23(A12D) and hFGF23‐WT was successfully achieved. As shown in Figure 1E, the ELISA assay showed that secreted hFGF23 was significantly increased in the overexpression cells; however, secreted hFGF23 in the hFGF23(A12D) group showed no significant difference from the control. Together, these observations suggest that both mutant hFGF23(A12D) and hFGF23‐WT are overexpressed in RC cells, but hFGF23(A12D) fails to be secreted.

### 3.2 Mutant hFGF23(A12D) inhibits proliferation ability of RC cells

Given that RC cells can be induced to differentiate into osteoblasts,^9^ we considered whether mutant hFGF23(A12D) would have an influence on RC cells. In order to determine the effect of mutant hFGF23(A12D) on cell proliferation ability, we performed the CCK‐8 assay in rLV‐mCMV‐ZsGreen, hFGF23(A12D) and hFGF23‐WT cells.

**Figure 2:**
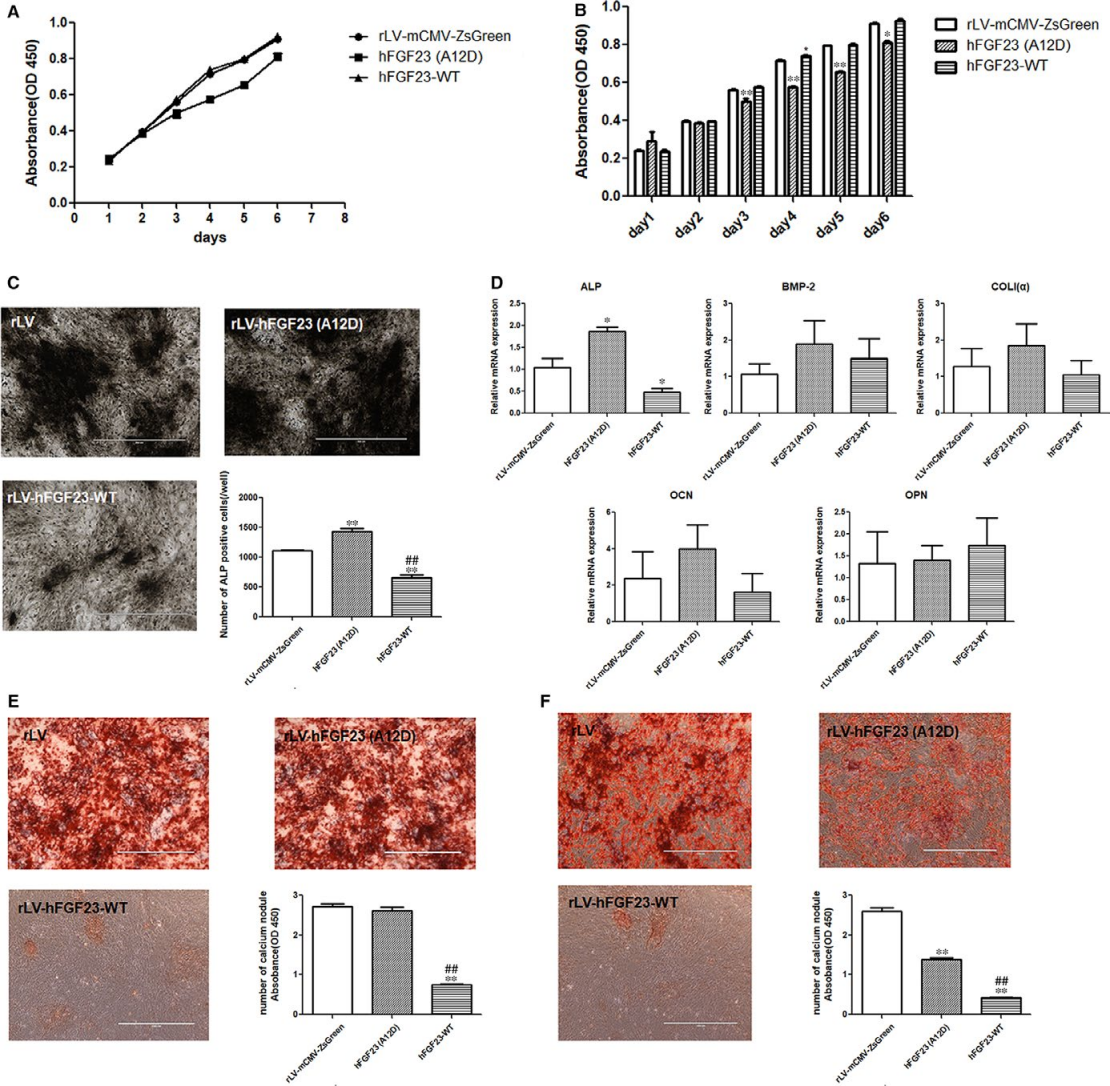
Mutant hFGF23(A12D) inhibits proliferation ability of RC cells, stimulates RC cell‐to‐osteoblast differentiation and inhibits matrix mineralization. A and B, CCK‐8 results show that hFGF23‐WT can slightly stimulate RC cells growth. C, ALP staining shows that mutant hFGF23(A12D) stimulates cell differentiation while hFGF23‐WT inhibits cell proliferation. D, RT‐PCR results show that mutant hFGF23(A12D) significantly increases ALP expression. E, Alizarin red staining was performed at day 21 and shows that the quantitation of mineralization nodules shows no significant alteration in the hFGF23‐A12D group and is significantly decreased in the hFGF23‐WT group. F, After osteoblasts were induced from RC cells, alizarin red staining was performed at day 21. The result shows that hFGF23(A12D) inhibits matrix mineralization compared to control group, while it increases matrix mineralization compared to hFGF23‐WT. *P < 0.05 and **P < 0.01 vs control group. ^##^ *P < *0.01 vs hFGF23(A12D) group. Values are presented as mean ± SD fold change

As shown in Figure 2A, on the first and second day, cell growth ability was not different among the three groups. From the third day up to the sixth day, cell proliferation was significantly decreased in the hFGF23(A12D) group, whereas the hFGF23‐WT group showed no significant difference from the control, except on day 4 when the proliferation of hFGF23‐WT cells was significantly higher than that of control cells (Figure 2B).

Taken together, these results indicate that mutant hFGF23(A12D) inhibits the proliferation ability of RC cells, while hFGF23‐WT does not. The inhibition could owe to cell transformation and differentiation (Figure S1).

### 3.3 Mutant hFGF23(A12D) stimulates differentiation from RC cells to osteoblasts and inhibits matrix mineralization

To address whether hFGF23(A12D) effects cell differentiation from RC cells to osteoblasts, we performed osteoblast induction in RC cells, including the rLV‐mCMV‐ZsGreen, hFGF23(A12D) and hFGF23‐WT groups. First, we analysed ALP activity in three groups by ALP staining. As shown in Figure 2C, the number of ALP positive cells was significantly increased in the hFGF23(A12D) group compared to the rLV‐mCMV‐ZsGreen control. In contrast, the number of ALP positive cells in the hFGF23‐WT group was significantly decreased compared to the rLV‐mCMV‐ZsGreen control. Subsequently, we detected a related marker in these induced osteoblasts by RT‐PCR (Figure 2D). The results showed that the expression of ALP was significantly increased in the hFGF23(A12D) group compared to the rLV‐mCMV‐ZsGreen control (approximately 1.9‐fold). The expressions of BMP‐2, Coli(α), OCN and OPN in the hFGF23(A12D) group were increased but showed no significant differences compared to the rLV‐mCMV‐ZsGreen group (BMP‐2: approximately 1.8‐fold, Coli(α): approximately 1.5‐fold, OCN: approximately 1.7‐fold).

Furthermore, we detected matrix mineralization by Alizarin red staining (stains calcium nodules) on day 21 in induced osteoblasts from these groups. As shown in Figure 2E, the number of Alizarin red‐positive calcium nodules was not significantly altered between the hFGF23(A12D) group and the rLV‐mCMV‐ZsGreen control group. However, the number of calcium nodules in the hFGF23‐WT group was significantly decreased compared to the rLV‐mCMV‐ZsGreen control. Moreover, we induced osteoblasts from RC cells and then infected osteoblasts with rLV‐mCMV‐ZsGreen, hFGF23(A12D) or hFGF23‐WT. As shown in Figure 2F, the number of Alizarin red‐positive calcium nodules in both the hFGF23(A12D) and hFGF23‐WT groups was significantly decreased compared to the rLV‐mCMV‐ZsGreen control. Moreover, the number of Alizarin red‐positive calcium nodules in the hFGF23(A12D) group was significantly increased compared to the hFGF23‐WT group. Together, these findings suggest that hFGF23(A12D) can stimulate differentiation from RC cells to osteoblasts and inhibit matrix mineralization. In contrast, hFGF23‐WT can inhibit both differentiation and matrix mineralization.

### 3.4 Mutant hFGF23(A12D) activates MAPK signalling pathway and increases FGFR3 expression during osteoblast differentiation in RC cells

**Figure 3:**
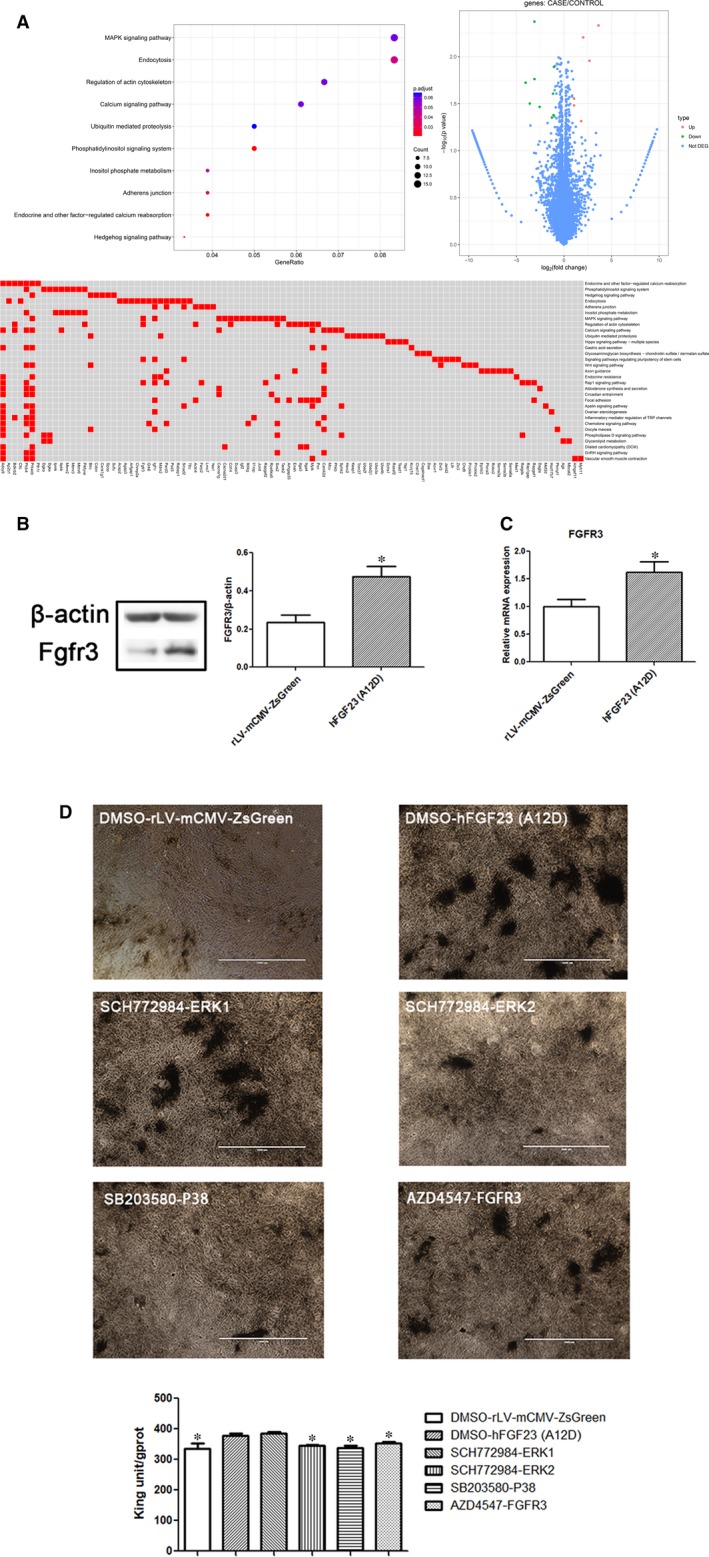
Mutant hFGF23(A12D) activates MAPK signalling and increases FGFR3 expression during osteoblast differentiation in RC cells. A, RNA‐sequence results show MAPK as the mainly related signalling pathway. B, Western blot and (C) RT‐PCR results show that FGFR3 expression is increased both at the protein level and mRNA level respectively. D, Infected cells maintained in the presence of SCH772984, SB203580 and AZD4547 or DMSO at day 5, and ALP staining was performed on day 15. The quantification of ALP shows that the ALP expression in the DMSO plus rLV‐mCMV‐ZsGreen, ERK2, p38 and FGFR3 groups is decreased. *P < 0.05 vs DMSO plus hFGF23(A12D) control group

In order to determine how mutant hFGF23(A12D) influences osteoblast differentiation, we performed RNA‐sequence analysis of the induced osteoblasts. As shown in Figure 3A and Table 2, compared to the control, the MAPK signalling pathway was enhanced significantly in the hFGF23(A12D) group, specifically involving Cacna1g/Cacna2d1/Cd14/Dusp3/Fgfr3/Igf1r/Igf2/Ikbkg/Il1rap/Jund/Prkacb/Rapgef2/Rps6ka5/Sos2/Taok2. Moreover, RNA‐sequencing results suggested that the expression of FGFR3 was significantly increased in the hFGF23(A12D) group compared to rLV‐mCMV‐ZsGreen. We performed western blot and RT‐PCR and confirmed the increase of FGFR3 (Figure 3B and C). Furthermore, we performed rescue experiments using MAPK‐ERK1/2, MAPK‐p38 and FGFR3 inhibitors. As shown in Figure 3D, the number of ALP positive cells was significantly decreased in the hFGF23(A12D) plus MAPK‐ERK2 inhibitor group, hFGF23(A12D) plus MAPK‐P38 inhibitor group and hFGF23(A12D) plus FGFR3 inhibitor group compared to the hFGF23(A12D) plus DMSO group. However, the number of ALP positive cells showed no significant difference in the hFGF23(A12D) plus MAPK‐ERK1 inhibitor group compared to the hFGF23(A12D) plus DMSO group.

**Table 2:** KEGG enrichment results of hFGF23(A12D) vs control group

ID	Description	GeneRatio	BgRatio	*P*‐value	*q*‐value	geneID	Count
rno04010	MAPK signalling pathway	15/180	300/8443	0.00183	0.05319	Cacna1g/Cacna2d1/Cd14/Dusp3/Fgfr3/Igf1r/Igf2/Ikbkg/Il1rap/Jund/Prkacb/Rapgef2/Rps6ka5/Sos2/Taok2	15
rno04144	Endocytosis	15/180	275/8443	0.00077	0.0364	Acap2/Agap3/Ap2b1/Arfgap1/Chmp2a/Cltc/Fgfr3/Grk6/Igf1r/Mdm2/Pard3/Psd3/Rabep1/Smad2/Tfrc	15
rno04810	Regulation of actin cytoskeleton	12/180	215/8443	0.00212	0.05319	Actn4/Arhgap35/Bdkrb2/Cd14/Enah/Fgfr3/Itga3/Itga4/Mylk/Pikfyve/Pxn/Sos2	12
rno04020	Calcium signalling pathway	11/180	189/8443	0.00233	0.05319	Adcy9/Bdkrb2/Cacna1g/Camk2d/Itpkb/Mcu/Mylk/Phkb/Plcb4/Prkacb/Sphk2	11
rno04120	Ubiquitin mediated proteolysis	9/180	141/8443	0.00312	0.05837	Btrc/Herc2/Keap1/Mdm2/Trim37/Ube2f/Ube2j1/Ube3a/Ube4b	9
rno04070	Phosphatidylinositol signaling system	9/180	98/8443	0.00023	0.01871	Dgka/Dgke/Ipmk/Itpkb/Mtmr2/Mtmr3/Mtmr6/Pikfyve/Plcb4	9
rno00562	Inositol phosphate metabolism	7/180	77/8443	0.00122	0.04169	Ipmk/Itpkb/Mtmr2/Mtmr3/Mtmr6/Pikfyve/Plcb4	7
rno04520	Adherens junction	7/180	73/8443	0.00089	0.0364	Actn4/Farp2/Igf1r/Lmo7/Pard3/Smad2/Yes1	7
rno04961	Endocrine and other factor‐regulated calcium reabsorption	7/180	56/8443	0.00017	0.01871	Adcy9/Ap2b1/Bdkrb2/Cltc/Plcb4/Prkacb/Pth1r	7
rno04340	Hedgehog signalling pathway	6/180	43/8443	0.00027	0.01871	Btrc/Cdon/Csnk1g1/Prkacb/Spop/Sufu	6

Together, these observations suggest that mutant hFGF23(A12D) activates the MAPK signalling pathway and increases FGFR3 expression during osteoblast differentiation from RC cells.

### 3.5 Mutant hFGF23(A12D) activates MAPK signalling pathway through interaction with intracellular domain of FGFR3

**Figure 4:**
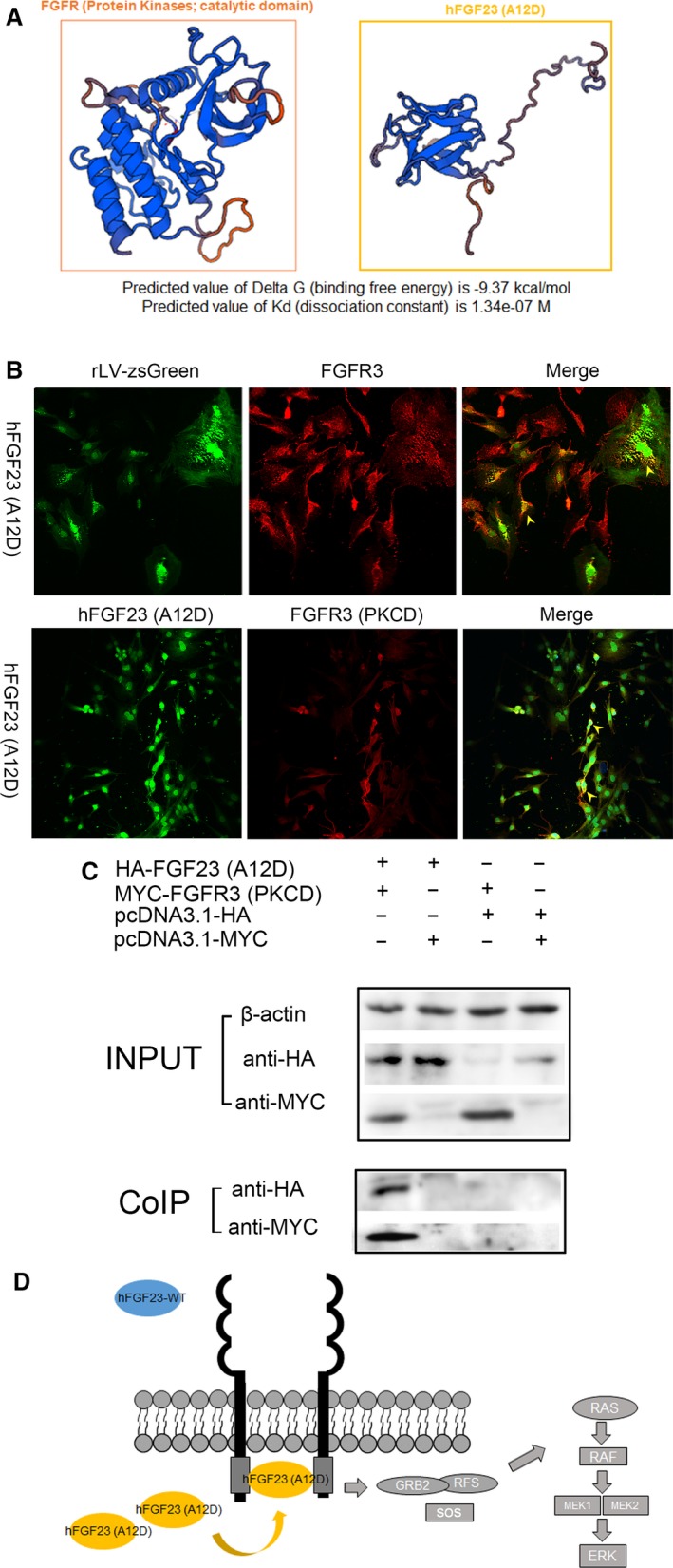
Mutant hFGF23(A12D) activates MAPK signalling through interaction with intracellular domain of FGFR3. A, Prediction results show that hFGF23(A12D) may bind to FGFR3(PKCD). B, Immunofluorescence staining shows the co‐localization of hFGF23‐WT/hFGF23(A12D) and FGFR3/FGFR3(PKCD). C, 293 T cells were transient transfection with HA‐FGF23(A12D)/pcDNA3.1‐HA and MYC‐FGFR3(PKCD)/pcDNA3.1‐MYC. Co‐immunoprecipitation results show that hFGF23(A12D) interacts with FGFR3(PKCD). D, The model illustrates that hFGF23(A12D) activates MAPK signalling through interaction with the intracellular domain of FGFR3

Given that our results showed that mutant hFGF23(A12D) can activate the FGFR3‐dependent MAPK signalling pathway, and mutant hFGF23(A12D) failed to be secreted by osteoblasts, we considered whether hFGF23(A12D) may function through the FGFR3 intracellular domain. First, we performed a bioinformatics analysis to predict whether the hFGF23(A12D) protein could bind to the intracellular domain of FGFR3. As shown in Figure 4A, results showed that the predicted value of Delta G (binding free energy) for the binding of the FGFR3 intracellular domain (PKCD) to hFGF23(A12D) is −9.37 kcal/mol, and the predicted value of Kd (dissociation constant) is 1.34e‐07 M, suggesting that hFGF23(A12D) may bind to the intracellular domain of FGFR3. Subsequently, immunofluorescence staining was performed with anti‐FGFR3 to identify whether hFGF23(A12D) interacts with FGFR3 in RC cells. Moreover, we transiently transfected RC cells with HA‐tagged hFGF23(A12D) and MYC‐tagged FGFR3(PKCD) and performed immunofluorescence staining with anti‐HA and anti‐MYC. As shown in Figure 4B, mutant hFGF23(A12D) was co‐localized with FGFR3, and hFGF23(A12D) was co‐localized with FGFR3(PKCD), suggesting that mutant hFGF23(A12D) may bind to the intracellular domain of FGFR3. Furthermore, we transiently transfected 293 T cells with HA‐tagged hFGF23(A12D) and MYC‐tagged FGFR3(PKCD), and used pcDNA3.1 with an HA‐tag and MYC‐tag as a control, to perform co‐IP. As shown in Figure 4C, hFGF23(A12D) interacted with FGFR3(PKCD) in 293 T cells.

Together, our results indicate that mutant hFGF23(A12D) activates the MAPK signalling pathway through interaction with the intracellular domain of FGFR3.

## 4 DISCUSSION

It is well known that hFGF23 belongs to a subfamily of mammalian endocrine FGFs, which can reduce phosphate reabsorption and suppress the production of 1,25(OH)2D through reduction of 1α‐hydroxylase.^13^ However, the function of mutant FGFs has not been clearly defined. Here, we clearly demonstrate that mutant hFGF23(A12D) stimulates the differentiation of RC cells to osteoblasts through the MAPK signalling pathway via FGFR3 activation, which interacts with hFGF23(A12D) through its intracellular cytoplasmic tyrosine kinase domain (Figure 4D). In this study, for the first time, we have identified a non‐secretory mutant hFGF23(A12D), which plays an important role in osteoblast differentiation.

Our results showed that mutant hFGF23(A12D) inhibited proliferation of RC cells and stimulated the differentiation of RC cells to osteoblasts. In contrast, a previous study demonstrated that wild‐type FGF23 can slightly increase cell proliferation but negatively regulates differentiation of osteoblasts through FGFR1c interactions.^9^ This suggests that the function of mutant hFGF23(A12D) during RC cell differentiation is different from wild‐type FGF23. We postulated that this is related to the mutation of A12D, which is located in the hydrophobic core of the signal peptide, and blocks hFGF23(A12D) secretion. Conversely, a previous study showed that hFGF23 also inhibited osteoblast mineralization through FGFR1c,^9^ while our results showed that compared to hFGF23, mutant hFGF23(A12D) less slightly inhibited mineralization in osteoblasts, which suggests that mutant hFGF23(A12D) is involved in osteoblast mineralization through a different pathway than hFGF23‐WT.

Our study also suggests that mutant hFGF23(A12D) inhibited proliferation of RC cells and stimulated the differentiation of RC cells to osteoblasts through its interaction with the intracellular domain of FGFR3. To date, most studies have revealed that secretory hFGF23‐WT functions by interacting with the extracellular domain of FGFRs, mostly in the presence of alpha‐Klotho.^14^, ^15^ For example, FGF23 binds to FGFR1c, FGFR3c and FGFR4 in vitro and the effect of FGF23 on vitamin D metabolism may be mediated through FGFR3.^16^ Study revealed that FGF23 can stimulate osteoblast proliferation and inhibit mineralization through FGFR1 in the presence of alpha‐Klotho.^10^ We predicted that the difference in secondary structure (Figure S4) between hFGF23‐WT and hFGF23(A12D) may result in novel functions. Therefore, we suggested that mutant hFGF23(A12D) can bind to certain intracellular signalling molecules. Notably, the results of bioinformatics analysis, co‐IP and immunofluorescence staining revealed that mutant hFGF23(A12D) interacts with the intracellular domain of FGFR3. Therefore, we have reason to consider that the non‐secretory mutant hFGF23(A12D) functions through intracellular action. FGFR3 is one of a family of four membrane‐bound tyrosine kinase receptors, largely expressed in cartilage and bone,^17^ consisting of an extracellular region, composed of three immunoglobulin‐like domains, a single hydrophobic membrane‐spanning segment and a cytoplasmic tyrosine kinase domain.^18^ Moreover, previous studies have demonstrated FGFR3 to be a negative regulator of bone growth, which can activate downstream signalling pathways.^19^ Of significance, FGFR3‐/‐ murine bones show severe and progressive bone dysplasia with enhanced and prolonged endochondral bone growth.^20^ In particular, FGFR3 inhibits chondrocyte proliferation in vitro through activating STAT‐1^21^ and also regulates the proliferation, osteogenic differentiation and matrix mineralization of BMSCs through ERK1/2 and p38 MAPK pathways.^22^ A study also suggests FGF23 binds to FGFR3 and then activates FGFR3, which may suppress chondrocyte proliferation through FGFR3.^14^, ^23^ Notably, the function of mutant hFGF23(A12D) is similar to the phenotype expressed by FGFR3 activation. Taken together, our results suggest that hFGF23(A12D) may stimulate differentiation from RC cells to osteoblasts through activating the MAPK signalling pathway through interactions with the intracellular domain of FGFR3.

Similar to heterozygous mice (FGF23+/‐), there were no significant differences in serum levels of FGF23 in MP patients. However, heterozygous mice showed no significant differences in appearance,^8^ while MP patients show no significant differences in appearance except the overgrowth of mandible compared to maxilla. Firstly, although a part of hFGF23‐WT is replaced by hFGF23 (A12D) in MP patients, MP patients are heterozygous and have normal expression of serum hFGF23 which result in the normal expression of serum phosphate and calcium and without severe symptom. Secondly, the mandible is a composite bone with endochondral growth at the condyle and intramembranous bone formation. Cartilage covers the surface of the mandibular condyle at the temporomandibular joint, and hyperplasia, hypertrophy and endochondral replacement do occur at this location. In contrast, the maxilla is mainly ossified at the membrane. Mutant hFGF23(A12D) interacts with the intracellular domain of FGFR3 in osteoblasts, which suggests that it may also have intracellular functions in chondrocytes. The different effects of hFGF23 (A12D) on chondrocytes and osteoblasts may cause the differences in growth between the mandible and maxilla, however, this requires further study.

In conclusion, we demonstrated that compared to hFGF23‐WT, mutant hFGF23(A12D) can stimulate differentiation of RC cells to osteoblasts through FGFR3‐mediated MAPK signalling pathway activation, and it can stimulate mineralization of osteoblasts, although mutant hFGF23(A12D) fails to be secreted from RC cells. Further understanding of hFGF23(A12D) may lead to better clinical treatments as well as new therapeutic developments to prevent mandibular prognathism, promote healing and optimize patient health.

## CONFLICTS OF INTEREST

There is no conflict of interest that could be perceived as prejudicing the impartiality of the research reported.

## AUTHORS’ CONTRIBUTION

Y. Tu, contributed to conception, design, acquisition, analysis, interpretation, drafted manuscript and critically revised manuscript; T. Qu, contributed to design, analysis, drafted manuscript; F Chen, contributed to conception, design and critically revised manuscript. All authors gave final approval and agrees to be accountable for all aspects of work ensuring integrity and accuracy.
