# Supplementary material for: Mutant hFGF23(A12D) stimulates osteoblast differentiation through FGFR3
Source: J Cell Mol Med. 2019 Feb 13;23(4):2933–42. doi: 10.1111/jcmm.14201 (PMC6433671; doi:10.1111/jcmm.14201)
Supplement: Supplementary file 1 [file JCMM-23-2933-s001.docx]

Supplementary

Figure S1. Real-time PCR results on the 4th day. The expressions of ALP, Coli(α) and RUNX2 in the hFGF23(A12D) group and in the hFGF23-WT group showed no significant differences compared to the rLV-mCMV-ZsGreen group.


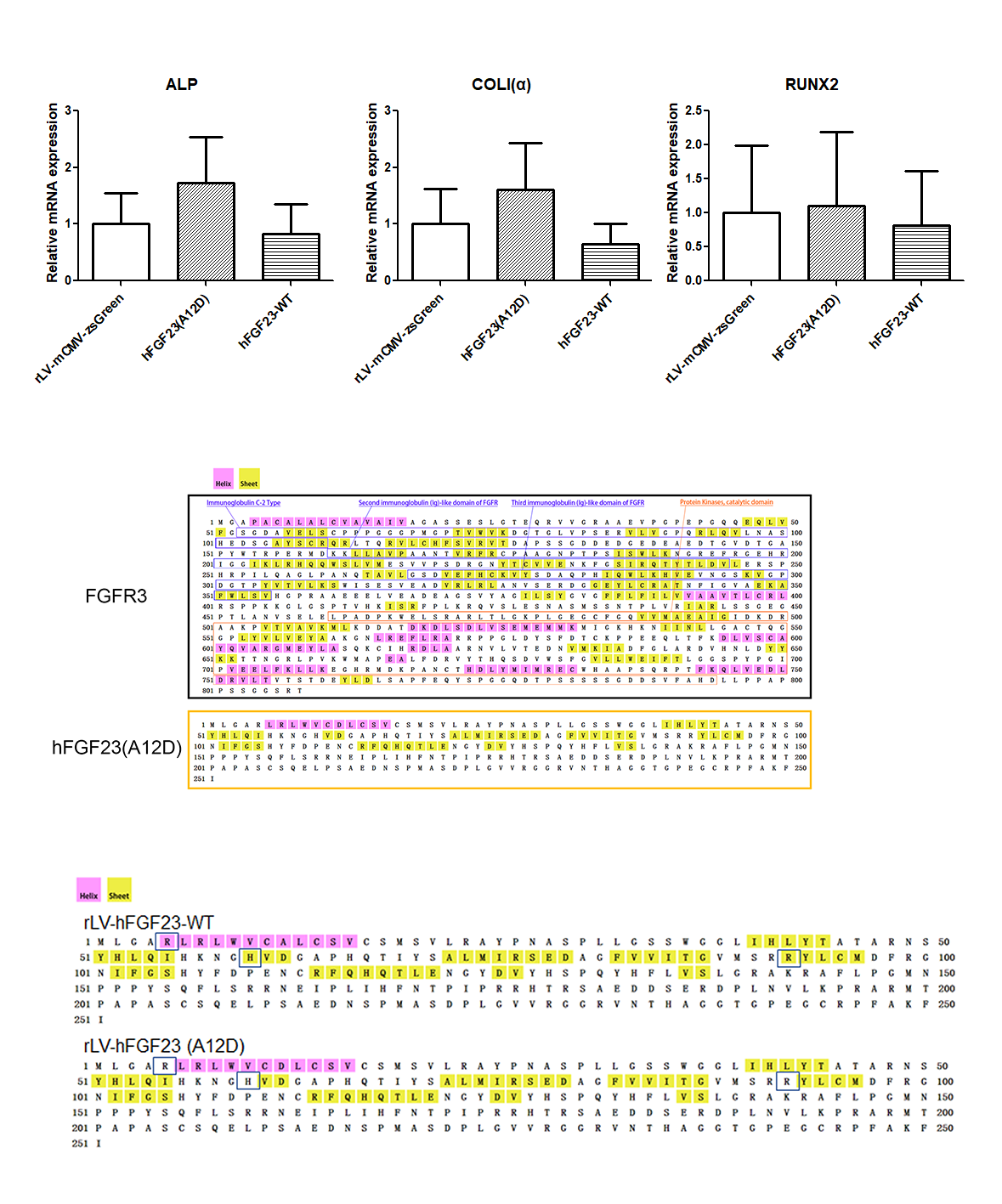


Figure S2. Western blot results on the 9th day. The expression of p-ERK1/2 and p-p38 in hFGF23(A12D) group is increased compared to the rLV-mCMV-ZsGreen group.


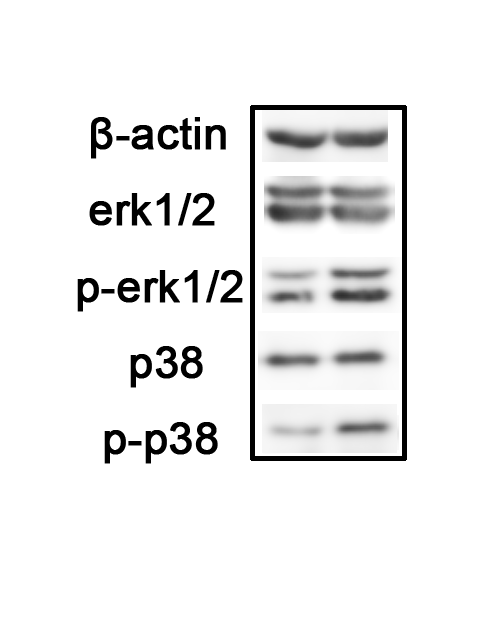


Figure S3. The secondary structure of FGFR3 and hFGF23(A12D).


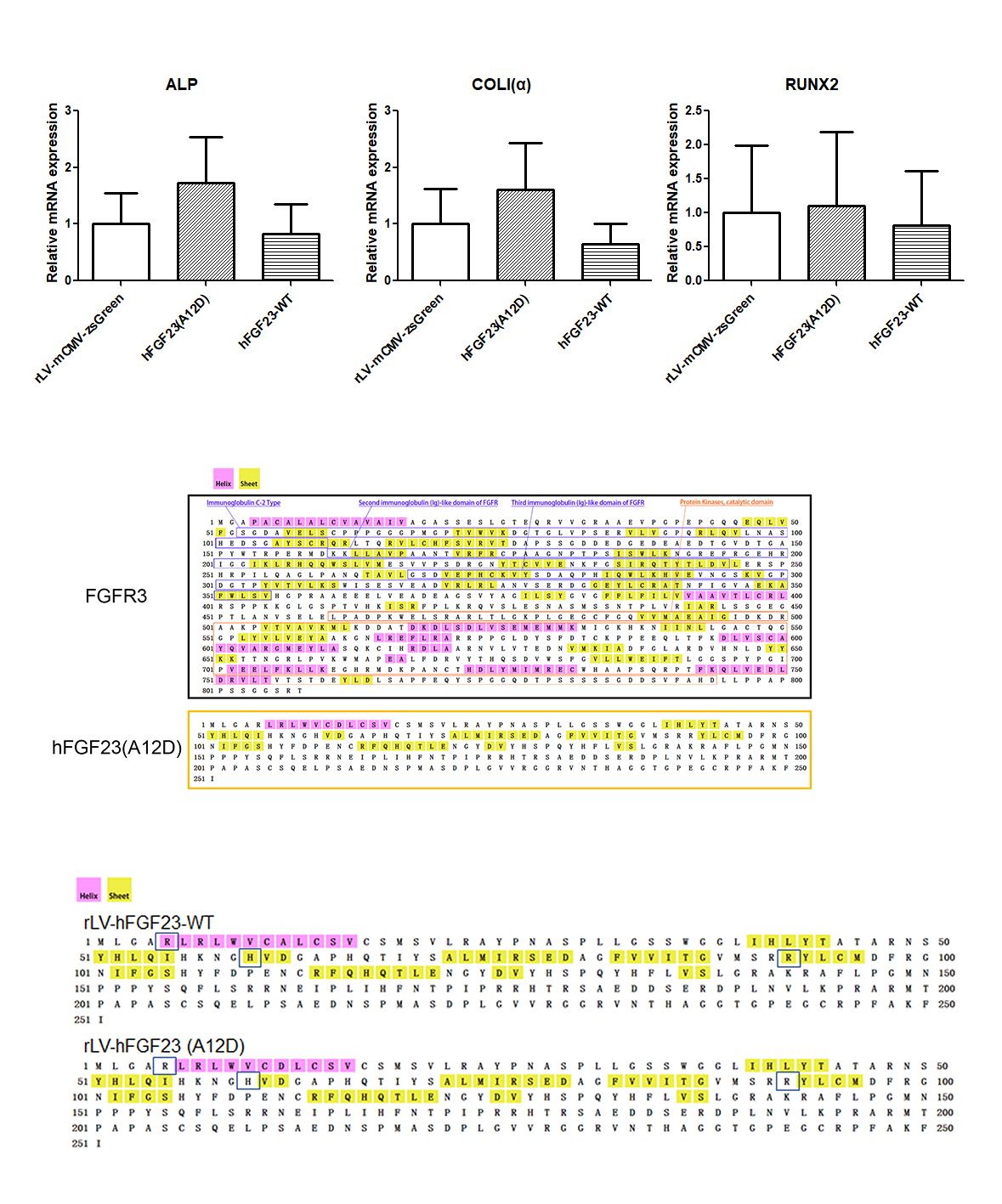


Figure S
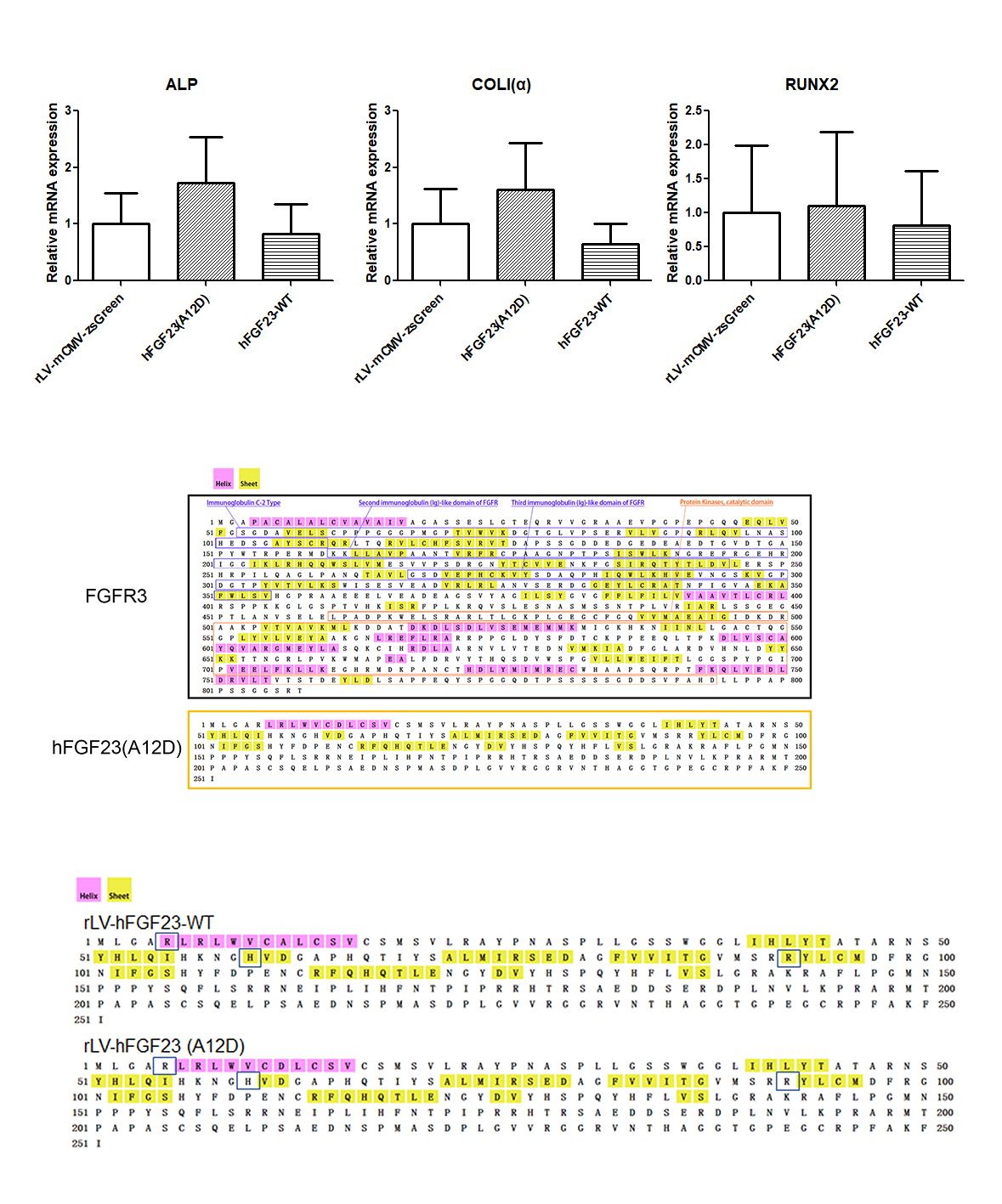
4. The prediction secondary structure of hFGF23 and hFGF23(A12D).
